# Editorial Announcement

**Published:** 1916-10

**Authors:** 


### EDITORIAL ANNOUNCEMENT

Copies are needed to complete files as follows: Sept., Oct. and Dec. 1914. All issues of 1915 except July and Oct. Jan., Feb. and Mar. 1916. Reserving the right to refuse offers beyond the number needed, we will allow three issues of the present or future, applied in any way desired, for each old number received. For example, you can extend your own subscription period, or have the journal sent with your compliments to a friend—and with no fear of having him annoyed by solicitations for continuance.

